# Language and rigour in qualitative research: Problems and principles in analyzing data collected in Mandarin

**DOI:** 10.1186/1471-2288-8-44

**Published:** 2008-07-10

**Authors:** Helen J Smith, Jing Chen, Xiaoyun Liu

**Affiliations:** 1 International Health Group, Liverpool School of Tropical Medicine, Liverpool, UK; 2 School of Public Health, Chongqing Medical University, Chongqing, PR China; 3 School of Public Health, Fudan University, Shanghai, PR China

## Abstract

In collaborative qualitative research in Asia, data are usually collected in the national language, and this poses challenges for analysis. Translation of transcripts to a language common to the whole research team is time consuming and expensive; meaning can easily be lost in translation; and validity of the data may be compromised in this process. We draw on several published examples from public health research conducted in mainland China, to highlight how language can influence rigour in the qualitative research process; for each problem we suggest potential solutions based on the methods used in one of our research projects in China.

Problems we have encountered include obtaining sufficient depth and detail in qualitative data; deciding on language for data collection; managing data collected in Mandarin; and the influence of language on interpreting meaning.

We have suggested methods for overcoming problems associated with collecting, analysing, and interpreting qualitative data in a local language, that we think help maintain analytical openness in collaborative qualitative research. We developed these methods specifically in research conducted in Mandarin in mainland China; but they need further testing in other countries with data collected in other languages. Examples from other researchers are needed.

## Background

In collaborative qualitative research in Asia, data are usually collected in the national language, and this poses challenges for analysis. [1] Translation of transcripts to a language common to the whole research team costs time and money; and meaning is easily distorted or lost in translation: in some languages and dialects there are literally no direct translations for some words and for other words several meanings can be assigned [2]. These problems are accentuated in qualitative studies carried out in mainland China, in collaboration with people for whom English is their first language, to be published in international English language journals. The grammatical structure of Mandarin differs substantially to English language which means the narrative of an interview might not be captured accurately [3,4].

We have encountered several problems to do with language when collecting, analysing and reporting data in Mandarin. In this paper we use examples from several published research papers to illustrate the challenges posed; in particular we focus on the implications of language and interpretation on rigour in qualitative research. We propose possible solutions to these problems, based on sound methodological principles that we have used in our subsequent research to help maintain rigour in the research process.

To illustrate the potential solutions we draw on specific examples from a descriptive study of directly observed therapy for administration of TB drugs to outpatients in the community in Chongqing Municipality; the findings are published elsewhere and ethics approval was granted by Chongqing Medical University[5]. The study aimed to identify ways that the TB health service delivery could be improved. We employed mixed methods: a survey to measure the level of direct observation by health workers, health facility patient record analysis to estimate treatment completion rates, and qualitative methods to explore patient and provider views on factors influencing adherence. For the qualitative component we conducted in-depth interviews in Mandarin to find out from patients and doctors about reported adherence, their views on direct observation, and factors that might influence adherence. Our research was directly relevant to the TB control programme in China, so we used the principles of Framework analysis [6], commonly used for applied or policy relevant qualitative research where the questions are clearly defined and objectives are set in advance. Framework is a systematic matrix based approach with distinct stages that allow transparent data management and interpretation. As with any qualitative analysis approach, the traditionally manual processes of data reduction (coding, searching, retrieving and sorting data) can be facilitated by using specialist software. We decided to use MAXqda to manage our data because most team members were experienced in using the software, and it is possible to import transcripts in Chinese characters (on computers with Chinese language support installed). Our research team comprised two social scientists (one with qualitative expertise), a postgraduate public health student, a medically trained tuberculosis expert, a statistician and a medically trained epidemiologist.

In the next section we describe four specific problems we have encountered conducting qualitative health research in mainland China, and for each problem we suggest potential solutions.

## Depth and detail in qualitative data

### Problems

Collecting qualitative data using individual interviews or focus group discussions requires considerable skill; too little direction and the participant will digress creating a high 'dross rate'[4]; ask too many short prompting questions and the interview turns into a structured questionnaire. Problems with question structure and flow, use of prompts, probes, 'directiveness' and non verbal feedback can usually be addressed through pilot testing and careful interviewing technique [7,8].

But there are special cultural issues to consider when conducting qualitative research in mainland China. For example, certain population groups are more likely to resist a researcher's prompt for detail and personal experiences. In a recent evaluation of a workplace intervention to provide family planning services to migrant workers in Shanghai, we found qualitative interviews yielded short responses that lacked depth and detail and interviewers found it difficult to encourage open dialogue [9]. In post-research debriefing discussions, the research team agreed that there could be several reasons for this. Most important was an appreciation of the unique circumstances of the participant – in this case young, unmarried female migrants who are considered a marginalised population in host cities – was crucial to understanding the reasons why they chose not to express their views openly. The team also considered that the prevailing culture of courtesy in mainland China may have prevented participants from being openly critical about people or services, particularly those in authority; this has been documented elsewhere in Asia [10]. This means that participants may conform to socially expected behaviour rather than disclose personal viewpoints [11].

### Suggested solutions

To help ensure our interviews yielded rich and detailed data, in our subsequent TB research we paid particular attention to the development and pilot testing of topic guides. We jointly developed topic guides in English for in depth interviews. This allowed for specialist input to the structure of the interviews, ensured relevant topic areas were covered, and that the types of questions asked were suitable for the target population. Topic guides were translated and pilot tested in Mandarin, Southwest dialect (a variant of standard Mandarin with different pronunciation). The interviewer was a postgraduate student from Chongqing and fluent in Southwest dialect.

An English speaking social scientist observed the conduct of the pilot interviews, with some simultaneous translation. This allowed for useful feedback on the use of probing questions and prompts to facilitate the conversation, observation of body language and active listening. The pilot interview recordings were transcribed in Mandarin, and to ensure transcript quality, the first few were translated and checked by the social scientists in the team. A brief 'eyeballing' of a transcript in any language where questions and responses are clearly marked can usually detect the balance of narrative between interviewer and interviewee, pick up on short responses and lack of probing, and sometimes identify where leading questions have been used.

It is difficult to negate the cultural barriers to open dialogue with interview participants in China. Most participants are naturally hesitant about sharing opinions, even more so when the interviewer probes for details about sensitive issues, such as illness. The patients interviewed as part of our study were at various stages of treatment for active tuberculosis, and some were initially unwilling to talk about their illness experience. We found that a good approach was to take time to build up trust with the participant, and de-personalise the questions so that participants felt they were not necessarily talking about themselves when they responded. As the student interviewer gained confidence in using these and probing and prompting questions, we found patients were more likely to discuss issues important to them in adhering to TB treatment; the resulting transcripts provided a rich and detailed narrative.

## Language and data collection

### Problems

The use of interpreters in international public health research is advocated by some, and different models are proposed for conducting interviews in this way [12]; but the impact of the interpreter on the research process needs consideration. Having an interpreter translate the researcher's questions directly can interrupt the flow of conversation and be distracting for the respondent and interviewer; while a more active model that allows the interpreter to carry out the interview means the researcher must relinquish control of the interview. In some cross-national research, 'tactical sampling' is employed to actively seek out key informants able to converse in English; researchers claim this avoids problems with interpretation, translation and miscommunication [1].

Simultaneous translation during interviews or focus group discussions can work where infrastructure allows for real time translation [13]; but this is dependent on the translator's skill and knowledge of the local dialect of the study population. An alternative is to translate all transcripts into a language common to the whole team after data is collected, but the risk of misinterpretation, misunderstanding and loss of a respondent's intended meaning is high unless the translator is familiar enough with the dialect to convey 'conceptual equivalence'[1]. This refers to the extent to which a term used in one language has a comparable meaning when translated into another language. Conceptual equivalence is particularly important in qualitative research collected in Mandarin, where some words have no linguistic equivalent in English or have more than one meaning [2]. Decisions made about translation can directly affect the accuracy of data collected and the validity of the research reported; researchers are therefore increasingly being encouraged to explain how translation was carried out, by whom, and how local meaning and cultural connotations are captured and reported in their data [14].

### Suggested solutions

One way to avoid problems of interpretation and ensure accurate meaning is captured during data collection is to conduct interviews and focus group discussions in the local language; this is greatly facilitated in a research team comprising bilingual researchers fluent in the local language (and dialect). Original words, phrases and concepts are securely embedded in context and the risk of misinterpretation and loss of participants' intended meaning is minimised. In our study, qualitative interviews were carried out by a postgraduate student fluent in Southwest dialect, and tape recorded with participant's permission. Recordings were transcribed verbatim in Chinese characters. Two Mandarin speaking researchers checked a sub-sample of transcripts against the original tape recordings to ensure local meanings were captured as far as possible.

## Managing data in Mandarin

### Problems

Important decisions during qualitative data analysis include: which sections of text to code, which data to retrieve and how, what search terms to use to explore the dataset, deciding which themes appear most important in understanding and explaining the phenomenon being studied, and how to explore and display relationships between themes. Traditional manual methods of cut and paste, filing and sorting of textual data can be slow to execute and difficult to describe accurately. When working as a team and with data across languages, we have found it is even more crucial to determine who is responsible for each component of the analysis.

In applied qualitative research it is important that the thematic framework used to code or index transcripts is informed by both the original topic guides and concepts emerging directly from the participants themselves. Identifying an initial coding frame requires researcher skill in pinpointing recurring themes and concepts, and developing meaningful labels for the data. When data are collected in a local language, those team members who are not fluent in this language are excluded from this process. On the other hand, collaborative working at this stage can prevent a profusion of inappropriate codes and arbitrary generation of emergent themes [15].

Teams working together across languages use various procedures to facilitate analysis – sometimes coding is completed in the local language and English summaries are provided for the whole team [16], others working with translated data may code and analyse all data in English [17]. In previous research in China we have used combinations of both, but usually where time is short the data coding, categorisation and identification of themes is done manually and in Mandarin, with discussion of important content of themes aided by English summaries [18]. We have found that in using these approaches it is difficult keep track of decisions made during the analysis, and even more difficult to describe exactly how it was done. Disclosure of qualitative analysis procedures is increasingly important [19], and 'audit' or 'decision' trails can help other researchers' judge for themselves whether the findings and interpretations are credible [20]. There is increasing recognition that computer software packages can help document these decisions, and ensure the processes of data reduction are visible, documented, retrievable and accessible [21].

### Suggested solutions

Based on our experience in the research with TB patients and providers, we recommend the coding framework is developed in the local language by more than one researcher, and is subsequently made available and discussed in a language common to the research team (in this case English). In our study, two bilingual researchers read through the Mandarin transcripts and independently listed recurring viewpoints relevant to the areas of questioning, and identified common themes emerging from the responses. Doing this independently allowed for more possibilities and ideas about relevant and meaningful code words. Consensus on a final thematic framework was reached through discussion.

**Figure 1:**
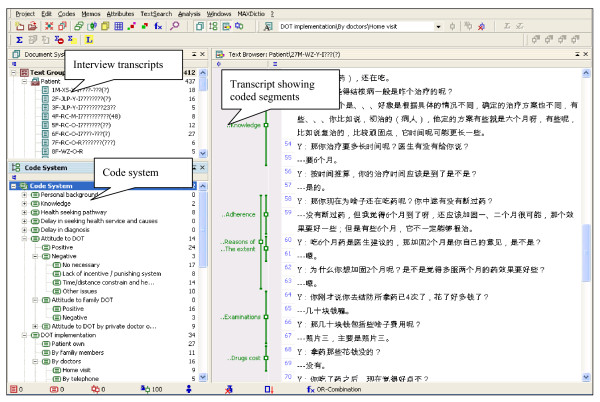
Screenshot of MAXqda.

We established a coding system in MAXqda based on the thematic framework. At the time the version of MAXqda we used (version 2) allowed transcripts to be imported in Mandarin, but did not support direct text input using Chinese characters, so the coding system had to be set up in English. Figure 1 shows a screenshot of MAXqda with a list of imported transcripts, the code system in English, and a transcript with coded segments.

We quickly realised the advantage of having the coding system in English; any refinement of the thematic framework could easily be discussed by the whole team, and this facilitated joint decision making between bilingual and English speaking researchers on the categorisation of coded data. The thematic framework and codes were modified and added to as other important issues and viewpoints emerged. The end product was a single database containing all coded interviews (using English labels as shown in figure 1) that could be viewed and accessed by the whole team.

**Figure 2:**
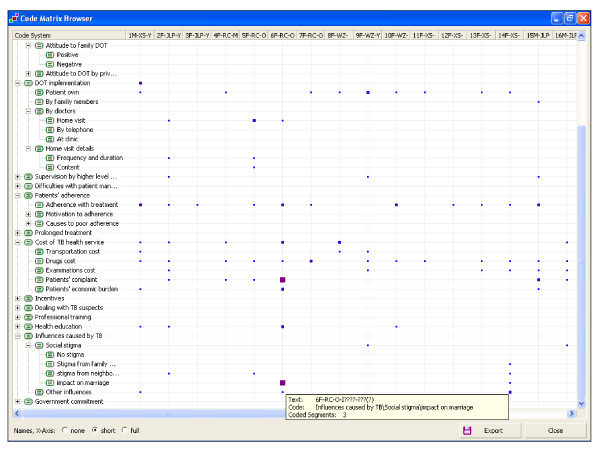
Code matrix browser.

Making sense of qualitative data requires systematic re-organisation and ordering of data chunks, allocating meaning and detecting patterns. MAXqda offers several tools for browsing and searching coded segments of text, which we found useful when working collaboratively across languages. In our study, a bilingual and an English speaking researcher explored the entire dataset by first using the 'code matrix browser' (see figure 2). This shows the frequency of use of codes across selected interviews; the size of the square shows how often the code was applied. Despite the English speaking researcher not being able to familiarise herself with the original data and the actual statements made by participants, this matrix helped both researchers determine and discuss which concepts were common across interviews, and identify codes that were used infrequently. We also browsed the data using the 'code relation browser', a matrix showing the concurrent use of codes. This helped identify where similar concepts were discussed together and where two or more codes could be collapsed into an overarching category.

## Language and interpreting meaning

### Problems

Working in teams to conduct qualitative research can increase rigor in analysis and encourage richer interpretation [22], but there are few examples of how teams working over geographic, cultural and linguistic distance actually achieve this. Social scientists will often recognise different patterns, meanings and interpretation in qualitative data to disease experts or epidemiologists, and local researchers are more likely to be familiar with the intricacies of the health system and socio-cultural characteristics of participants than those from outside. Bringing these differing perspectives to bear on emerging conceptual frameworks and explanations helps ensure the findings are grounded in and supported by the data and accurate underlying meaning (and conceptual equivalence) is conveyed.

It is our experience from previous research in mainland China that recognising patterns and being able to interpret the data accurately can be the most challenging part of the process and can involve lengthy discussion about the meaning of informant accounts. For example, in a qualitative study exploring women's views of obstetric care in government hospitals in Shanghai we found that traditional beliefs influenced women's decisions to avoid Caesarean section as it would damage their 'yuan qi'. There is no English equivalent of 'yuan qi', which refers to inherited energy of the body. We consulted together as a research team to make sure the meaning of women's statements was clear and not misinterpreted in the English write up [23]. Similarly, in the study of young female migrants' views of family planning services, a main theme was related to privacy in obtaining services [9]. Interviewees frequently used the term 'bu hao yi si'; again there is no literal translation but, after much discussion about the appropriate meaning in English, we agreed to refer to it as 'feeling uncomfortable or embarrassed', and quoted the Mandarin phrase in the final publication.

The decision to publish in a local or international journal is dependent on the research question, funding requirements and judgements about the policy and practice implications of the research. Publication in a language other than that which the data were collected and analysed clearly affects the researcher's ability to accurately convey the meaning of the data, particularly through verbatim quotes; and some argue that translation of direct quotations conceals culturally-loaded meanings [1]. These are decisions an international collaborative research team must consider.

### Suggested solutions

Data interpretation is frequently described as an intuitive and imaginative process which cannot be reduced to simple mechanical steps [24], but we found this process can be more critical, the interpretations more valid, and the findings more credible, by involving researchers with a) different methodological perspectives and disciplinary interests, b) detailed understanding of the study context including the cultural characteristics of participants and the structure of the health system, and c) the ability to accurately convey meaning of data collected in a local language. The process of seeing patterns in qualitative data and drawing out meaning is made more thorough by involving the entire research team, taking advantage of their differing disciplinary, language and cultural perspectives. In our study of TB treatment in Chongqing, we established a system that enabled this.

After identifying categories of data, we summarised data relevant to each category in a matrix by case, using translated extracts and direct quotes in English. A bilingual postgraduate student, who also conducted the interviews, translated the extracts and quotes, and these were independently translated by a bilingual social scientist; any disagreements were resolved by discussion or involvement of another bilingual researcher. These English summaries formed the basis of discussions about patterns in the data, comparisons of individual accounts and experiences, and alternative plausible explanations of the themes. For each of the categories, English and bilingual researchers looked across the data and explored the range of attitudes and experiences of sub-groups (i.e patients and doctors, male and female, new and re-treated patients, and across counties). One surprising and important outcome of these discussions was being able to clarify that patient reports of expensive treatment costs did not refer to the cost of anti-tuberculosis treatment (which is provided for free), but to the cost of additional traditional Chinese medicines.

The literature relating to rigor in qualitative research suggests researchers should display enough data to allow a judgement on whether the interpretations are supported by the data and the conclusions are justified [15,25]. Our research on TB was written up as a policy brief for circulation in China and in English for an international peer reviewed journal. We used verbatim quotes in Mandarin together with English translations to illustrate the meaning of each main theme so that Mandarin and English speaking readers could judge for themselves the credibility of our interpretations and research findings [5].

## Discussion

Descriptive qualitative analysis is an iterative process, with the aim of meaningfully re-classifying codes into categories and themes. Published examples of qualitative research conducted in local languages sometimes do not describe the analysis process adequately; it is often difficult to discern in what language data were analysed, how coding frames were developed and codes derived, and how consensus was reached on analysis and interpretation. Some researchers devise their own ways of assuring data quality, analysing data in the local language using translated summaries of relevant text extracts [26], and we believe these should be made explicit.

We have described methods we used that allowed us to collect detailed qualitative data in Mandarin, manage that data effectively, produce plausible data categories and arrive at a meaningful interpretation of our data. This level of teamwork across languages was made possible by using a matrix based approach that allowed each stage of the analysis to remain visible to all researchers, and software that facilitated browsing and retrieval of relevant data. We are aware that these may not be the only solutions to the problems highlighted in this paper, and obstacles remain even within our proposed methods.

We found qualitative analysis software useful for keeping track of decisions made during analysis and for sharing the project coding and categorisation between the whole team. We are aware that there are limitations with most software packages, for example, the language restrictions in MAXqda (version 2) meant we underutilised the text search functions available. However, we are aware that the most recent version (MAXqda2007) and other software programmes such as NVivo 7 now support coding and searching in any language including Unicode [27] character languages such as Chinese. We developed data matrices and charts using Microsoft Word, but MAXqda2007 now includes a facility to construct data matrices, tables and other visual models [28]. In addition, the founders of the Framework approach, the National centre for Social Research, have just launched their own software with matrix capabilities, specifically for use alongside the Framework approach [29].

We accept that cultural issues may only be partly responsible for the problems we encountered in obtaining depth and detail in qualitative interviews. Another important consideration is capacity in interviewing technique. Although the use of qualitative data collection methods in public health research is growing, medical and public health training in universities in mainland China emphasises epidemiological methods, and qualitative methods receive limited consideration. We believe capacity in qualitative health research will develop as researchers begin to recognise the contribution of this approach. Better integration of basic and social science research is needed in health systems and health services research, and better collaboration between researchers across cultures is important in achieving this, particularly in countries like China. International collaborative programmes present the opportunity to acquire research methods expertise and disease specific knowledge that can be applied to public health priorities in mainland China, and this will encourage greater participation of Chinese researchers [30]. However, due to the language barrier, cultural differences, and difficulties in applying the research methods across languages, Chinese researchers may face obstacles to participating in global research programmes, and particularly in qualitative health research. We think the methodological principles outlined in this paper contribute to the growing consensus on acceptable methods for conducting qualitative health research across languages and cultures.

We are aware that in our example we relied on English summaries to form the basis of team discussions about patterns in the data, explanations of themes and interpretation. We emphasise that these translated extracts are to aid discussion; often interpreting the data requires the research team returning to individual transcripts to clarify meaning and concepts and make sure that data categories are reasonable and emerging themes are meaningful. In various research projects we have found that this stage in the analysis is the most time consuming.

When qualitative research conducted in one language is written up in another (for example in English for publication in international journals) in our experience it makes sense to provide at least some of the data as illustrative quotes in the local language. Depending on the journal, this may or may not be straightforward. Public health journals whose editors are used to publishing epidemiological research sometimes have restricted word limits. On the other hand, this can be easier in journals operating an open access policy, or who publish primarily online, as they often allow for additional tables or files.

## Conclusion

We have described how language can influence rigour in the qualitative research process, using examples specifically from public health research conducted in mainland China. We have suggested methods for overcoming problems associated with collecting, analysing, and interpreting qualitative data in a local language, that we think help maintain analytical openness in collaborative qualitative research. We developed these methods specifically in research conducted in Mandarin in mainland China; they require further testing and evaluation in other countries with data collected in other languages. We agree that analysis and 'seeing meaning' in qualitative data is inherently collaborative [31], and have demonstrated where computer software can open the possibilities to do this when working in teams and across languages. Examples from other researchers conducting collaborative qualitative research internationally are needed.

## Competing interests

The authors declare that they have no competing interests.

## Authors' contributions

HJS conceived of and wrote the paper in discussion with JC and XL. All authors contributed to the design of data collection tools in the initial research study cited in reference 5. JC collected the data for that study as part of a Masters thesis, and conducted the analysis with HJS and XL. All authors contributed to revising the final manuscript.

## Pre-publication history

The pre-publication history for this paper can be accessed here:



## References

[B1] Mangen S (1999). Qualitative research methods in cross-national settings. International Journal of Social Research Methodology.

[B2] Tsai JH-C, Choe JH, Lim JMC, Acorda E, Chan NL, Taylor V, Tu S-P (2004). Developing culturally competent health knowledge: Issues of data analysis of cross-cultural, cross-language qualitative research. International Journal of Qualitative Methods.

[B3] Twinn S (1998). An analysis of the effectiveness of focus groups as a method of qualitative data collection with Chinese populations in nursing research. Journal of Advanced Nursing.

[B4] Twinn S (1997). An exploratory study examining the influence of translation on the validity and reliability of qualitative data in nursing research. Journal of Advanced Nursing.

[B5] Hu D, Liu X, Chen J, Wang Y, Wang T, Zeng W, Smith H, Garner P (2008). Directly observed therapy for tuberculosis in a Province in China: a descriptive study. Health Policy & Planning.

[B6] Ritchie J, Spencer L, O'Connor W, Ritchie J, Lewis J (2004). Carrying out qualitative analysis. Qualitative research practice.

[B7] Britten N (1995). Qualitative interviews in medical research. British Medical Journal.

[B8] Hope A, Timmel S (1984). Training for transformation: A handbook for community workers.

[B9] Qian X, Smith HJ, Huang W, Zhang J, Huang Y, Garner P (2007). Promoting contraceptive use among unmarried female migrants in one factory in Shanghai: a pilot workplace intervention. BMC Health Services Research.

[B10] Jones E, Bulmer M, Warwick DP (1983). The courtesy bias in Southeast Asian surveys. Social Research in Developing countries.

[B11] Bin Liang, Hong Lu (2006). Conducting fieldwork in China: Observations on collecting primary data regarding crime, law and the criminal justice system. Journal of Contemporary Criminal Justice.

[B12] Pitchforth E, van Teijlingen E (2005). International public health research involving interpreters: a case study from Bangladesh. BMC Public Health.

[B13] Esposito N (2001). From meaning to meaning: the influence of translation techniques on non-English focus group research. Qualitative Health Research.

[B14] Birbili M (2000). Translating from one language to another. Social Research Update.

[B15] Richards T, Richards L, Kelle U (1995). Using hierarchical categories in qualitative data analysis. Computer Aided Qualitative Data Analysis: Theory, Methods and Practices.

[B16] Tolhurst R, Zhang T, Yang H, Gao J, Tang S (2004). Factors affecting the implementation of health legislation and its impact on the rural poor in China: A case study of implementation of the maternal and infant health care law in two poor counties. Int J Health Plann Mgmt.

[B17] Raven J, Chen Q, Tolhurst R, Garner P (2007). Traditional practices and beliefs in the postpartum period in Fujian province, China: a qualitative study. BMC Pregnancy & Childbirth.

[B18] Qian X, Smith H, Liang H, Liang J, Garner P (2006). Evidence-informed obstetric practice during normal birth in China: trends and influences in four hospitals. BMC health Services Research.

[B19] Anfara VA, Brown KM, Mangione TL (2002). Qualitative analysis on stage: Making the research process more public. Educational Researcher.

[B20] Murphy E, Dingwall R, Greatbatch D, Parker S, Watson P (1998). Qualitative research methods in health technology assessment: a review of the literature. Health Technol Assess.

[B21] Basit TN (2003). Manual or electronic: the role of coding in qualitative data analysis. Educational Research.

[B22] Barry CA, Britten N, Barber N, Bradley C, Stevenson F (1999). Using reflexivity to optimise teamwork in qualitative research. Qualitative Health Research.

[B23] Xu Qian, Helen Smith, Li Zhou, Ji Liang, Paul Garner (2001). Evidence based obstetrics in four hospitals in China: An observational study of women's preferences and provider's views. BMC Pregnancy & Childbirth.

[B24] Ritchie J, Lewis J, Eds (2003). Qualitative research practice.

[B25] Spencer L, Ritchie J, Lewis J, Dillon L Quality in qualitative evaluation: a framework for assessing research evidence. Government Chief Social Researcher's Office Occasional Papers Series No2.

[B26] Tolhurst R, Zhang T, Yang H, Gao J, Tang S (2004). Factors affecting the implementation of health legislation and its impact on the rural poor in China: A case study of implementation of the maternal and infant health care law in two poor counties. International Journal of Health Planning and Management.

[B27] Unicode homepage. http://www.unicode.org/.

[B28] MAXqda homepage. http://www.maxqda.com/.

[B29] National Centre for Social Research. http://www.natcen.ac.uk/natcen/pages/hw_framework.htm#formats.

[B30] Department for International Development China: Development research priorities. Report on consultations for DFID's global research strategy 2008–13. http://www.dfid.gov.uk/research/China-Consultation-Report.pdf.

[B31] Richards L (1999). Qualitative teamwork: Making it work. Qualitative Health Research.

